# Increased Uptake of Silica Nanoparticles in Inflamed Macrophages but Not upon Co-Exposure to Micron-Sized Particles

**DOI:** 10.3390/cells9092099

**Published:** 2020-09-15

**Authors:** Eva Susnik, Patricia Taladriz-Blanco, Barbara Drasler, Sandor Balog, Alke Petri-Fink, Barbara Rothen-Rutishauser

**Affiliations:** 1 Adolphe Merkle Institute, University of Fribourg, Chemin des Verdiers 4, 1700 Fribourg, Switzerland; eva.susnik@unifr.ch (E.S.); patricia.taladrizblanco@unifr.ch (P.T.-B.); barbara.drasler@unifr.ch (B.D.); sandor.balog@unifr.ch (S.B.); alke.fink@unifr.ch (A.P.-F.); 2 Department of Chemistry, University of Fribourg, Chemin du Musée 9, 1700 Fribourg, Switzerland

**Keywords:** nanoparticles, silica, lipopolysaccharide, endocytosis, stimulation, inflammation, co-exposure, macrophages

## Abstract

Silica nanoparticles (NPs) are widely used in various industrial and biomedical applications. Little is known about the cellular uptake of co-exposed silica particles, as can be expected in our daily life. In addition, an inflamed microenvironment might affect a NP’s uptake and a cell’s physiological response. Herein, prestimulated mouse J774A.1 macrophages with bacterial lipopolysaccharide were post-exposed to micron- and nanosized silica particles, either alone or together, i.e., simultaneously or sequentially, for different time points. The results indicated a morphological change and increased expression of tumor necrosis factor alpha in lipopolysaccharide prestimulated cells, suggesting a M1-polarization phenotype. Confocal laser scanning microscopy revealed the intracellular accumulation and uptake of both particle types for all exposure conditions. A flow cytometry analysis showed an increased particle uptake in lipopolysaccharide prestimulated macrophages. However, no differences were observed in particle uptakes between single- and co-exposure conditions. We did not observe any colocalization between the two silica (SiO_2_) particles. However, there was a positive colocalization between lysosomes and nanosized silica but only a few colocalized events with micro-sized silica particles. This suggests differential intracellular localizations of silica particles in macrophages and a possible activation of distinct endocytic pathways. The results demonstrate that the cellular uptake of NPs is modulated in inflamed macrophages but not in the presence of micron-sized particles.

## 1. Introduction

In recent decades, the use of nanotechnology has increased in many industrial applications [1]. Among different nanomaterials (i.e., materials with any external dimensions in the nanoscale, 1–100 nm) [2], silica (SiO_2_) nanoparticles (NPs) have attracted considerable attention in various applications due to their appealing physicochemical properties [3,4]. Based on the structure defined by X-ray-diffraction spectroscopy [5], there are two main forms of silica: amorphous and crystalline. Amorphous silica, with its tunable properties and stability, is a promising candidate for biomedical applications, such as drug delivery and molecular imaging [6,7], and could be intentionally introduced into the human body for disease treatments and diagnosis [8]. In addition, amorphous silica is used in the food industry to prevent poor flow in viscous products or “caking” in powdered products [9]. It also can be used as a thickener in pastes, as a carrier of flavors, and as a means to clarify beverages and control foaming [10,11,12].

Widespread applications of different silica NPs may increase their chances of co-exposure to human beings in daily life. For this reason, a deep understanding of combined NP exposures in a complex environment, such as the human body, is crucial. Many uptake studies have investigated the effect of one SiO_2_ NP type [13,14,15] or its combination with other molecules [16,17], whereas studies on the combined effects of silica particles with different physicochemical properties, i.e., sizes, on human cells are lacking. It has been demonstrated that some NPs can be internalized faster when co-exposed due to the activation of multiple parallel endocytic pathways [18,19,20]. As an example, Li et al. [21] showed that there is a strong interplay between different-sized silica NPs on their cellular uptakes in HeLa cells. The uptake of 50-nm silica NPs co-exposed with 100-nm silica NPs was significantly greater than in single exposures, indicating that the presence of bigger particles greatly promoted the uptake of smaller ones.

Another important aspect is to study the NPs’ interactions in an inflamed microenvironment in the human body. The immune system is the primary target reacting to NPs from the local environment [22]. Phagocytic immune cells, such as macrophages, are one of the first cells exposed to foreign particles and are able to initiate the innate immune response [23,24]. In addition, macrophages are able to integrate multiple signals (i.e., stimuli) from their local microenvironment and respond by the production of inflammatory cytokines [24,25,26]. Depending on the stimuli, macrophages can be activated (i.e., polarized) into different phenotypes. Lipopolysaccharide (LPS) and interferon-gamma (IFN-γ) polarize them towards the proinflammatory (M1) phenotype involved in host defense and produce tumor necrosis factor alpha (TNF-α), nitric oxide, interleukin (IL)-12, and IL-23. On the other hand, the exposure of macrophages to the IL-13, IL-10, or IL-4 cytokines induces an anti-inflammatory (M2) phenotype, producing IL-10, IL-1, and transforming growth factor beta (TGF-β) [25,26]. Humans are regularly exposed to LPS-producing Gram-negative bacteria. It has been demonstrated that bacterial infections and the presence of a high LPS concentration (above 1 pg/mL) in the bloodstream can provoke severe inflammatory reactions [27,28]. Some inflammatory stimuli can also reprogram the macrophage endocytic pathways, enabling the internalization of larger amounts of NPs [29,30,31]. Therefore, it is beneficial to understand the interaction of multiple NPs with inflamed macrophages and their impacts on the uptake activity.

To elaborate on the existing knowledge, and since we can be exposed to multiple particles and inflammatory stimuli at the same time, we examined the combined effect of two different-sized silica particles on macrophage cellular uptake after stimulation with LPS. The particles were administered after the LPS stimuli sequentially and simultaneously for different time points. We confirmed that, in LPS prestimulated cells, there was an increase in the uptake of both sizes of silica particles. However, we did not observe differences in the particle uptake under co-exposure conditions compared to single exposures.

## 2. Materials and Methods

### 2.1. Chemical and Reagents

All chemicals and reagents were purchased from Sigma-Aldrich (Buchs, Switzerland), while all cell culture reagents were purchased from Gibco, Thermo Fisher Scientific (Zug, Switzerland), unless stated otherwise.

### 2.2. Synthesis and Characterization of Particles

Synthesis of SiO_2_ particles (i.e., nanometer- and micrometer-sized particles) labeled with fluorescent dyes was performed following the Stöber method [32], as previously reported in the literature [33]. For 59-nm SiO_2_-BDP FL (i.e., BODIPY^®^-fluorescein) particles, a mixture of 6.75 mL of water (Milli-Q), 104 mL of absolute ethanol (EtOH) (VWR, Dietikon, Switzerland), and 3.9 mL of 25% ammonium hydroxide (NH_4_OH) (Merck, Zug, Switzerland) were heated at 60 °C in a 500-mL rounded-bottom flask provided with a reflux system. After 30 min, 11 mL of tetraethyl orthosilicate (TEOS) were added to the mixture using a plastic syringe. Finally, after 2 min, a mixture of 200 µL of BODIPY FL *N*-Hydroxysuccinimide (NHS) ester (10 mg/mL in dimethyl sulfoxide (DMSO), Lumiprobe, Hunt Valley, MD, USA) and 4 µL of (3-aminopropyl) triethoxysilane (APTES) were added to the flask. The reaction was further heated for 4 h, cooled down to room temperature, and dialyzed (0.2-μM cellulose acetate filter) against Milli-Q water for five days and stored in the dark at 4 °C.

SiO_2_-Cy5 (i.e., Cyanine 5) particles (920 nm) were prepared as the 59-nm SiO_2_-BDP FL particles with slight modifications. Then, 12.9 mL of Milli-Q, 14.7 mL of EtOH, and 16 mL of NH_4_OH were placed in a polystyrene bottle provided with a magnetic bar and heated to 40 °C using a water bath. After 30 min, 8.4 mL of TEOS were quickly added to the bottle. The reaction was stirred for 15 min, leading to the formation of SiO_2_ NPs with a hydrodynamic diameter of ~492 nm. The dispersion was then transferred to a 500-mL plastic bottle and heated at 40 °C, followed by the addition of 22.38 mL of water, 8.43 mL of EtOH, and 2.15 mL of NH_4_OH after temperature stabilization. After 10 min, 16 mL of TEOS were added to the mixture. The addition of TEOS was repeated four times. Two minutes after the fourth addition of TEOS, 300 µL of a mixture containing 1.15 mg of Cy5-NHS ester (Lumiprobe) and 3.9 µL of (3-aminopropyl) triethoxysilane (APTES) in DMSO were added to the bottle. The reaction was further heated for 45 min, cooled down to room temperature, and centrifuged twice at 1000 rpm for 15 min to remove the excess of unbounded Cy5. Particles were redispersed in Milli-Q water and stored in the dark at 4 °C.

The particles were characterized by transmission electron microscopy (TEM, FEI Technai G2 Spirit, Thermo Fisher Scientific, Waltham, MA, USA) equipped with a Veleta CDD camera (Olympus, Tokyo, Japan), and their core diameter was calculated using a FIJI particle size analysis (ImageJ, National Institutes of Health, Bethesda, MD, USA). The hydrodynamic diameters of SiO_2_ particles in Milli-Q and in complete medium were recorded with a dynamic light scattering (DLS) spectrometer (LS Instruments AG, Fribourg, Switzerland) at the scattering angle of 90° and laser wavelength 660 nm. To calculate dimensions of SiO_2_ particles, the obtained correlation functions were analyzed according to method described previously [34,35]. In order to measure the colloidal stability of SiO_2_ particles, we performed DLS measurements of both particles in Milli-Q and in complete medium at the incubation time 24 h at 37 °C. The polydispersity index and ζ-potential were determined by dynamic light scattering and phase-amplitude light scattering (ZetaPALS) in Milli-Q water (Brookhaven 90Plus Particle Size Analyzer, Brookhaven Instruments Corp., Holtsville, NY, USA), respectively. Ten DLS and zeta potential measurements were recorded for each sample to estimate the mean and the standard deviation. The particle concentration was assessed by measuring dry weights of particle suspensions after water evaporation at 70 °C.

### 2.3. Cell Culture

The murine macrophage cell line J774A.1 was purchased from American Type Culture Collection (ATCC, Rockville, MD, USA). Cells were cultivated in Roswell Park Memorial Institute 1640 (RPMI, Gibco, Life Technologies Europe B.V., Zug, Switzerland) cell culture medium, supplemented with 10% fetal bovine serum (FBS), 1% penicillin/streptomycin, and 1% l-glutamine, all *v*/*v*, referred as the complete cell culture medium (cRPMI). Cells were grown in culture flasks with the surface area 150 cm^2^ (TRP, Trasadingen, Switzerland) and kept at 37 °C, 5% CO_2_, and 95% relative humidity. Cells were visually observed under a phase-contrast light microscope (Motic AE2000, Moticam BTU 10, Motic, Wetzlar, Germany) coupled to a camera (Moticam BTU 10) for their confluence, morphology, and viability. New cell passage was prepared at 70–80% confluence. Used cell culture medium was removed, and cells were washed with PBS to remove dead cells in the suspension. Fresh cRPMI (20 mL) was added to the flask. Adhered cells were harvested carefully using a cell scraper (Sarstedt, Sevelen, Switzerland). Cell suspension was diluted in a fresh cRPMI (1:8 ratio) and transferred into a new culture flask for further experiments.

### 2.4. Cell Seeding

Murine macrophages J774A.1 were maintained in cell culture flasks in cRPMI (passage numbers 5 to 10). When cells reached 80% confluence, they were rinsed with PBS and detached from the wells using a cell scraper in 10-mL cRPMI. The cell concentration was determined by an automated cell counter (EVE, NanoEnTek Inc., Seoul, South Korea) via the trypan blue (0.4% *v*/*v* in PBS) exclusion methods. For confocal laser scanning microscopy, cells were seeded in an 8-well glass bottom u-Slide (Cat. No. 80827, ibidi, Graefelfing, Germany), with a growth area 1 cm^2^ per well and a cell suspension volume of 300 µL. Cells were seeded at a density of 52,000 cells/cm^2^ or 52,000 cells/300 µL (corresponding to 170,000 cells in 1 mL). For flow cytometry, cells were seeded in a 6-well flat bottom cell culture plate (Cat. No. 354118, Corning, Reinach, Switzerland), with a growth area 9.6 cm^2^ per well and medium volume 3 mL. Cell density was 52,000 cells/cm^2^ or 500,000 cells/3 mL (corresponding to 170,000 cells in 1 mL). Cells were incubated at 37 °C, 5% CO_2_, and 95% relative humidity for 24 h before exposure to LPS or particles.

### 2.5. Exposures to Silica Particles

#### 2.5.1. Pretreatment of Cells with LPS

J774A.1 cells were cultured for 24 h in fresh cRPMI in the presence or absence of 1-μg/mL LPS (*Escherichia coli* strain O111:B4, Cat. No. L4391, Sigma-Aldrich). The cell supernatants were then collected and stored at −80 °C for cytokine release (ELISA) and at 4 °C for membrane rupture (lactate dehydrogenase (LDH)) assays.

#### 2.5.2. Sequential Particle Exposure

Suspension of 59-nm SiO_2_-BDP FL and 920-nm SiO_2_-Cy5 particles was first prepared in Milli-Q water at the concentration 1 mg/mL. Before the experiments on cells, the suspension was diluted in cRPMI to reach the final concentration of 20 µg/mL. After 24-h incubation with LPS, cells were rinsed 3 times with PBS and exposed to 920-nm SiO_2_-Cy5 particles at a concentration of 20 µg/mL in cRPMI. After 4-h exposure to 920-nm SiO_2_-Cy5 particles, the excess of external particles was removed. Cells were rinsed with PBS, exposed to 59-nm SiO_2_-BDP FL NPs at a concentration of 20 µg/mL in cRPMI, and kept in the incubator for 24 h. Particles were administered to the cells via a premixed method (i.e., a single particle type was added to cRPMI immediately prior to the cell exposure) in order to ensure homogenous particle deposition on the cells.

#### 2.5.3. Simultaneous Particle Exposure

Suspension of 59-nm SiO_2_-BDP FL and 920-nm SiO_2_-Cy5 particles was prepared at the concentration of 1 mg/mL in Milli-Q and then diluted in cRPMI to the final concentration of 20 µg/mL. After 24-h incubation with LPS and rinsing 3 times with PBS, cells were exposed to both particles at the same time (i.e., simultaneously) for either 4 h or 24 h in the incubator. Particles were added in a premixed manner (both particles were mixed in cRPMI prior to the cell exposure) at a final concentration of 20 µg/mL.

#### 2.5.4. Controls

To determine the effect of LPS on macrophages, we used unstimulated cells grown in cRPMI for 24 h. Cells unexposed to silica particles served as a second control. Instead of particles, Milli-Q was added to cRPMI at the same volume as used for particle exposures. Cells exposed to only 920-nm SiO_2_-Cy5 or 59-nm SiO_2_-BDP FL particles for 4 h or 24 h in a premixed manner served for the investigation of impact of individual particle types (single-exposure controls).

### 2.6. Confocal Laser Scanning Microscopy

After the predefined exposure times (i.e., 4 h or 24 h), cells grown and exposed in the 8-well µ-slides were rinsed 3 times with PBS and fixed with 4% paraformaldehyde (PFA; in PBS, *w*/*v*) for 15 min at room temperature. After rinsing 3 times with PBS, cells were permeabilized with 0.1% TritonX-100 (*v*/*v*; in PBS) for 15 min at room temperature. Next, cells were rinsed 3 times with PBS and stained with rhodamine-phalloidin (λ_ex_ at 540 nm and λ_em_ at 565 nm; Molecular Probes, Life Technologies, Switzerland) in PBS (1:100 dilution; stock concentration 6.6 µM) to stain the F-actin cytoskeleton for 1 h at room temperature in the dark. Then, the staining solution was discarded, and the cells were rinsed 3 times with PBS. For nuclei staining, 4′,6′-diamidino-2-phenylindole (DAPI) (λ_ex_ at 340 nm and λ_em_ at 488 nm) in PBS was added to the cells (1:50 dilution; stock concentration 100 µg/mL). After rinsing, samples were stored in fresh PBS at 4 °C until analysis. 

Image acquisition was performed with an inverted Zeiss LSM 710 Meta microscope (Axio Observer.Z1, Zeiss, Feldbach, Switzerland) equipped with a 405-nm excitation diode (DAPI) and 488-nm (BDP FL), 561-nm (rhodamine), and 633-nm (Cy5) lasers. The analysis was performed using a Plan-Apochromat 63×/NA 1.4-immersion oil objective lens (Zeiss GmbH, Jena, Germany).

Two-dimensional projection images (XY step size of 0.13 µM) with corresponding z-stacks (step size of 0.42 µM) were acquired at 63× magnification to confirm the uptake of the fluorescently labeled particles. After acquisition, the stacks were analyzed using FIJI software (ImageJ). To quantify the degree of colocalization between both particles (fluorophores), the Pearson correlation coefficient (PCC) was determined from the corresponding confocal images using Imaris software version 9.5 (Bitplane, Oxford Instruments, Zürich, Switzerland).

### 2.7. Analysis of Colocalization between SiO_2_ Particles and Lysosomes

Cells were co-exposed to 20 μg/mL of the 59-nm SiO_2_-BDP FL NPs and 920-nm SiO_2_-Cy5 sequentially or simultaneously for 4 h and 24 h. After exposure, the cells were washed three times with PBS and further incubated with fresh cRPMI supplemented with 75-nM LysoTracker Red DND-99 (Invitrogen, Thermo Fisher Scientific Inc., Waltham, MA, USA) for 1 h. Afterwards, the supernatant containing LysoTracker was removed, and cells were washed with PBS and fixed with 4% PFA in PBS (pH 7.4). For nuclei staining, 4′,6′-diamidino-2-phenylindole (DAPI) in PBS was added to cells (1:50 dilution; stock concentration 100 µg/mL). After rinsing, samples were stored in PBS at 4 °C until analysis. Images were obtained by confocal laser scanning microscopy Zeiss LSM 710 (Axio Observer.Z1) with a 63× oil-immersion objective using a 405-nm laser for DAPI, 488-nm laser for SiO_2_-BDP-FL NPs, 561-nm laser for LysoTracker Red, and 633-nm for SiO_2_-Cy5 particles. The co-localization was calculated using the imaging analysis software Imaris.

### 2.8. Flow Cytometry

For flow cytometry, cells grown in 6-well cell culture plates were used. Cell supernatant was collected from each well in a 6-well plate and stored at 4 °C for the LDH assay analysis. Cells were gently rinsed 1 time with PBS, avoiding possible detachment. Incubation of cells in 1 mL of Accutase^TM^ (Cat. No. 00-4555-56, Thermo Fisher Scientific) for 15 min at 37 °C in combination with scraping was used for detaching adherent cells. Cell suspensions from each well with the same treatment conditions were combined and transferred into polystyrene test tubes. The cell concentration was determined by automated cell counter via the trypan blue (0.4% *v*/*v* in PBS) exclusion methods. Cell supernatants were centrifuged for 5 min at 300× *g*. Cell-free supernatant was discarded, and the cell pellet was resuspended in cold flow cytometry (FC) buffer, containing 1% bovine serum albumin (BSA) and 1-mM ethylenediaminetetraacetic acid (EDTA) in PBS (pH 7.4). In order to distinguish between live and dead, cells were stained on ice for 10 min with DAPI at the final concentration of 0.5 µg/mL. After the addition of 3 mL of fresh FC buffer, cells were centrifuged for 5 min at 300× *g*. Cell pellets were resuspended in 0.5 mL of fresh FC buffer for the flow cytometer analysis (BD LSRFortessa^TM^, BD Biosciences, San Jose, CA, USA). Before sample acquisition, cell suspension was filtered through a 40-µM cell strainer (Corning). Cells were kept on ice throughout the staining procedure until the analysis. 

Data were acquired using BD LSRFortessa^TM^ (BD Biosciences, Allschwil, Switzerland) and analyzed using FlowJo software (Version 10.6.2, TreeStar, Woodburn, OR, USA).

### 2.9. Cell Viability/Membrane Rupture Assay

Cell supernatants were collected from wells and stored at 4 °C. Activity of the enzyme lactate dehydrogenase (LDH) in the supernatants was measured in triplicates according to the manufacturer’s protocols. The absorbance of the colorimetric product formazan was determined spectrophotometrically (Benchmark microplate reader, BioRad, Cressier, Switzerland) at 490 nm, with a reference wavelength of 630 nm, at 30-s intervals for ten measurements. The values were represented as fold increase of slopes (5–10 min reactions) of the tested samples relative to that of untreated controls.

### 2.10. Enzyme-Linked Immunoassay (ELISA)

The amount of released pro-inflammatory cytokine TNF-α was determined using a DuoSet ELISA Development Kit (R&D Systems, Zug, Switzerland) according to the supplier’s protocols. For one sample (LPS 24 h), the samples were diluted 1:5 (*v*/*v*) in reagent diluent to remain within the detection limit of the instrument. The measurements were performed in high-binding polystyrene 96-well plates (Corning). Standards and samples were run in triplicates. The concentrations of the TNF-α released in the cell culture medium were calculated based on the standard curves and fitted with a four-parameter logistic (4PL) approach using GraphPad Prism 8 software (GraphPad Software Inc., San Diego, CA, USA).

### 2.11. Statistical Analysis

All statistical analyses were performed in GraphPad Prism 8 software using an unpaired two-tailed *t*-test or one-way ANOVA. The results are reported as the mean with standard deviation of the mean (SD), averaged over three independent biological replicates. * denotes *p* ≤ 0.05.

## 3. Results

### 3.1. Characterization of Silica Particles

**Figure 1:**
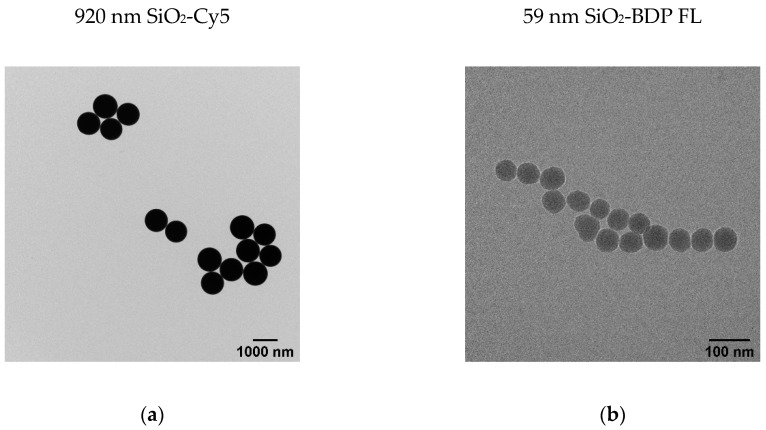
Characterization of the silica (SiO_2_) particles. (**a**) Representative transmission electron micrographs (TEM) of 920-nm-size SiO_2_-Cy5 (Cyanine 5) microparticles. (**b**) Representative TEM of 59-nm-size SiO_2_-BDP FL (BODIPY^®^-fluorescein) nanoparticles (NPs).

Fluorescently labeled SiO_2_ particles of two different sizes were synthesized following the Stöber method, as described in the Methods section. In order to determine particle size and the size distribution, we performed a transmission electron microscopy (TEM) analysis (Figure 1). The size distribution, determined by TEM, is presented as histograms in Supplementary Materials Figure S1. The data revealed an average core diameter (d_c_) of 920 ± 88 nm for SiO_2_-Cy5 and 59 ± 6 nm for SiO_2_-BDP FL NPs. The TEM analysis demonstrated sphere-like shapes of both SiO_2_ particles.

Table 1 summarizes the physicochemical characteristics of SiO_2_ particles, measured by TEM and DLS. An average hydrodynamic diameter (d_h_) of SiO_2_-Cy5 particles in water is 908 ± 13 nm and SiO_2_-BDP FL NPs is 76 ± 1 nm. When the particles were incubated in the presence of serum proteins (cRPMI containing 10% FBS), the average hydrodynamic diameters increased for both SiO_2_ particles. The zeta potential of both particles was negative. The polydispersity index (PDI) values range from 0.03 (for 920-nm SiO_2_-Cy5 particles) to 0.16 (for 59-nm SiO_2_-BDP FL NPs), indicating a monodisperse population with respect to the particle size. Results from DLS measurements of the hydrodynamic diameter revealed that both SiO_2_ particles are colloidally stable in Milli-Q and in cRPMI over 24 h of incubation time.

### 3.2. J774A.1 Macrophages Change Morphology after LPS Stimulation

**Figure 2:**
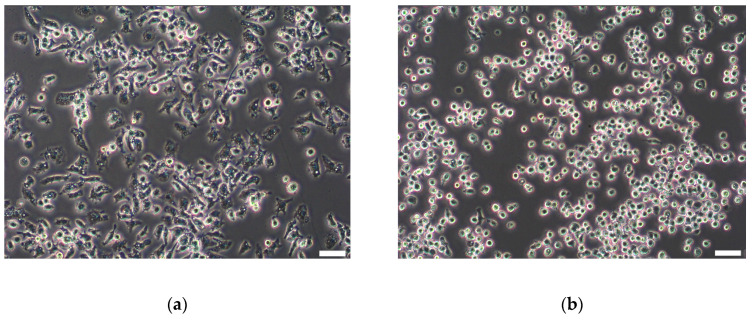
Representative light microscopy images of J774A.1 macrophages. (**a**) Macrophages 24 h after prestimulation with 1-µg/mL lipopolysaccharides (LPS). (**b**) Unstimulated macrophages. After stimulation, the change in cell morphology (larger cells with more protrusions) is visible in comparison to more round cells in the control culture. Scale bar: 100 µM.

To trigger a proinflammatory macrophage phenotype, J774A.1 cells were grown for 24 h and then stimulated with 1-µg/mL LPS for an additional 24 h. We expected morphological changes after LPS treatment as a sign of macrophage activation. J774A.1 cells treated with LPS showed changes in cell size, protrusions, and morphology (Figure 2a) in comparison to unstimulated cells (Figure 2b). Similar changes in the cell morphology after 24 h of LPS prestimulation were observed in all the passages, as shown in Figure S2.

### 3.3. Cytotoxicity of SiO_2_ Particles after Prestimulation of Cells with LPS

**Figure 3:**
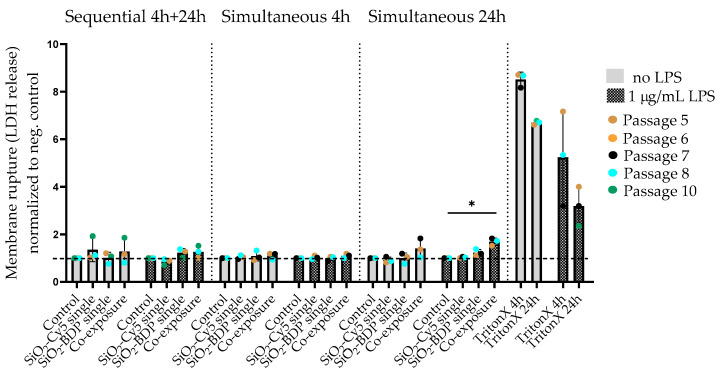
Cytotoxicity of LPS and SiO_2_ particles, determined by lactate dehydrogenase (LDH) assay. J774A.1 macrophages were prestimulated with 1-µg/mL LPS for 24 h and post-treated with a single particle type or used for sequential or simultaneous co-exposure. As a control, supernatants from non-LPS-stimulated cells were analyzed. Data was normalized to the negative control (dotted line) and expressed as a fold increase in LDH release over the negative controls. TritonX (0.2%) served as a positive control for membrane rupture. One-way ANOVA (GraphPad Prism) was used for the statistical significance analysis (* *p* < 0.05).

To mimic inflamed macrophages, the J774A.1 mouse macrophages were prestimulated with 1 µg/mL of LPS for 24 h; then, LPS was removed, and the cells were exposed to 920-nm SiO_2_-Cy5 microparticles or 59-nm SiO_2_-BDP FL NPs alone or their combination in a sequential and simultaneous manner. Control cells were only exposed to SiO_2_ particles without a pretreatment of macrophages with LPS. Figure 3 represents a fold change in the LDH release normalized to the negative control. For most conditions, no cytotoxic effects were observed. Additionally, cell prestimulations with LPS only showed no elevated levels of LDH in the supernatants. A moderate but significant increase in LDH release was only observed after 24 h of simultaneous SiO_2_ particle co-exposures in LPS prestimulated cells.

### 3.4. LPS Prestimulated J774A.1 Macrophages Show an Increase in TNF-α Secretion but No Elevation in the Presence of Particles 

The secretion of the proinflammatory cytokine TNF-α was assessed in LPS prestimulated J774A.1 macrophages, as well as after particle post-exposure (sequential and simultaneous), to assess a possible exacerbation of inflammatory effects.

**Figure 4:**
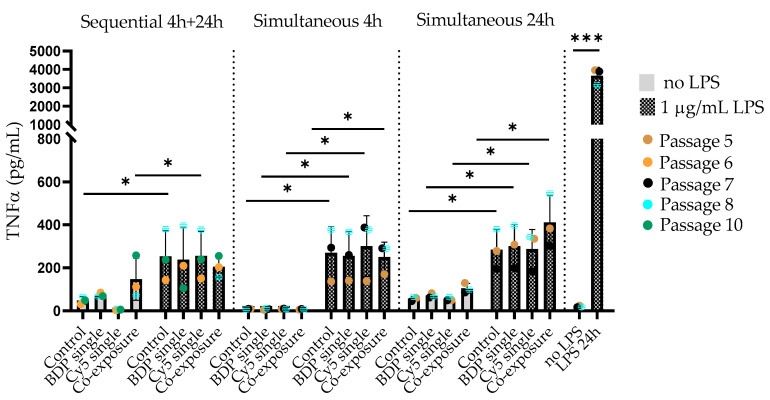
J774A.1 cells were prestimulated with 1-µg/mL LPS and post-exposed to SiO_2_ particles or only exposed to particles. To investigate the production or release of tumor necrosis factor alpha (TNF-α) in the cell culture media, we collected the supernatants and performed an enzyme-linked immunosorbent assay (ELISA). We have observed a significant increase in TNF-α production in the supernatants of LPS prestimulated cells compared to unstimulated cells (* *p* < 0.05 and *** *p* = 0.0002).

Figure 4 shows the TNF-α levels (in pg/mL) in unstimulated and LPS prestimulated J774A.1 cells exposed to SiO_2_ particles under different exposure conditions. The highest increase (*** *p* = 0.002) in TNF-α production (~3000 pg/mL) was observed after 24-h stimulation with 1-µg/mL LPS. When the LPS stimulus was removed from the cell culture medium and the cells were post-exposed to SiO_2_ particles, TNF-α was still detectable in the supernatants from LPS pre-exposed cells but to an approximately 10-times lower extent (~300 pg/mL). No difference between the control and particle-exposed cells was observed. Only particle-exposed cells showed no TNF-α release.

### 3.5. Cellular Distribution of SiO_2_ Particles in LPS Prestimulated J774A.1 Macrophages 

**Figure 5:**
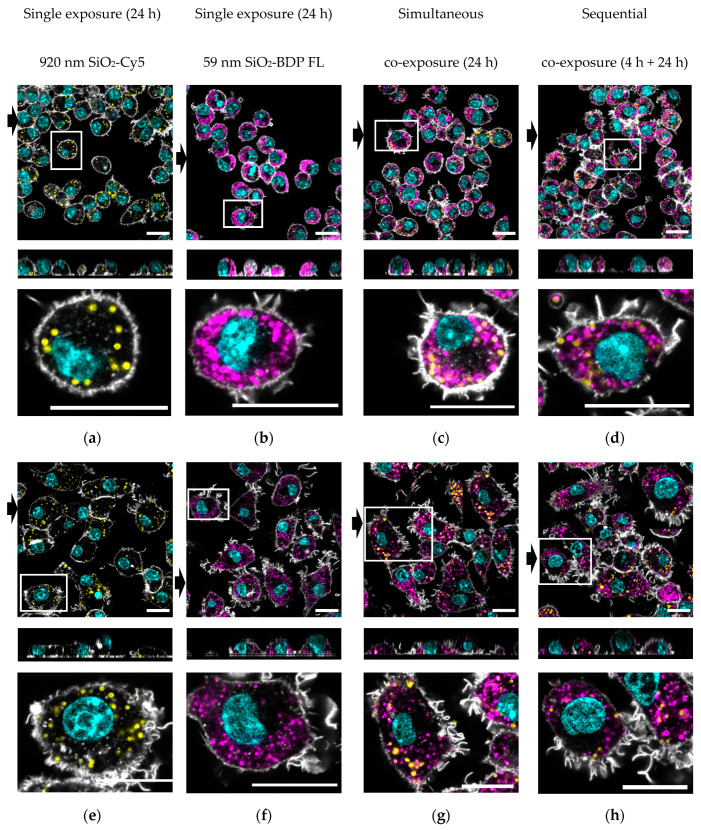
Representative confocal laser scanning micrographs with the corresponding xz projections demonstrate the uptake of 59-nm SiO_2_-BDP FL NPs and 920-nm SiO_2_-Cy5 particles in J774A.1 macrophages. Upper panels (**a**–**d**): Cells exposed to single 920-nm SiO_2_-Cy5 particles (yellow), single 59-nm SiO_2_-BDP FL NPs (magenta), or a combination of SiO_2_ particles simultaneously for 24 h or sequentially for 4 h and 24 h. Bottom panels (**e**–**h**): Cells were prestimulated with 1-µg/mL LPS for 24 h and then post-exposed to single 920-nm SiO_2_-Cy5 particles, single 59-nm SiO_2_-BDP FL NPs, or a combination of both particles simultaneously for 24 h and sequentially for 4 h and 24 h. F-actin was stained as the cytoskeleton marker using a rhodamine-phalloidin conjugate (grey), and the nucleus was stained using 4′,6-diamidino-2-phenylindole (DAPI) (cyan). The black arrows represent the position of the xz projection (at the bottom), showing the intracellular localization of the particles. Zoom-in images (shown under each representative image) clearly demonstrate the cellular distribution of two types of SiO_2_ particles. Scale bar: 20 µM.

The visualization of particle distribution in the cells was performed by confocal laser scanning microscopy. Figure 5 shows representative images of the fluorescent SiO_2_ particles’ cellular distribution after 24 h post-exposure.

It could be shown that, in both single- and co-exposure experiments, the unstimulated J774A.1 (Figure 5a–d) and LPS prestimulated cells (Figure 5e–h) show internalized particles after 24 h. The activated cell phenotype upon LPS treatment could be confirmed by F-actin staining, showing the more spread cell body and protrusions (Figure 5e–h). However, we could not observe the visual differences in particle uptake between the single- and co-exposure conditions. Representative images of the simultaneous exposures for 4h are provided in Figure S3.

Additional experiments with LysoTracker^TM^ dye (i.e., an organic heterotricyclic compound that accumulates within the acidic lumen of lysosomes) were performed in order to estimate the localization of different-sized SiO_2_ particles within lysosomes in unstimulated and LPS prestimulated cells. The images obtained with confocal laser scanning microscopy are shown as Figure S4a,b. We could observe a clear fluorescence overlap between 59-nm SiO_2_-BDP FL NPs and lysosomes in all co-exposure conditions for both unstimulated and LPS prestimulated cells. On the other hand, there was very little visual fluorescence overlap between 920-nm SiO_2_-Cy5 particles and lysosomes.

### 3.6. Distinct Intracellular Localization of Different-Sized SiO_2_ Particles in LPS Prestimulated J774A.1 Macrophages

**Figure 6:**
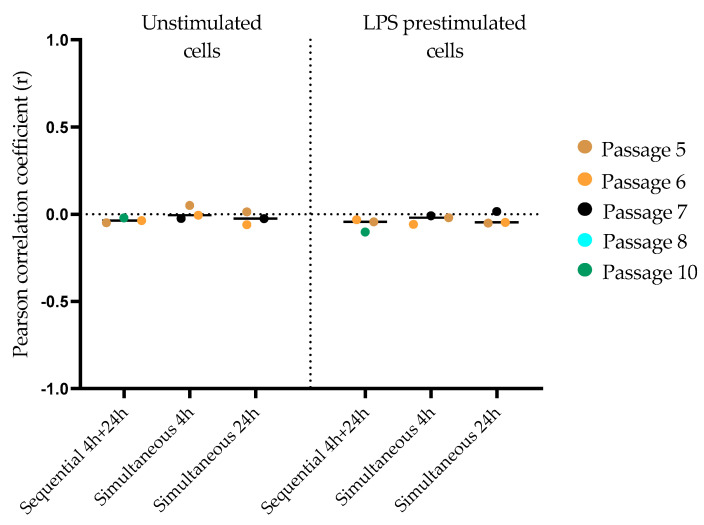
The colocalization between SiO_2_ particles of different sizes was analyzed using Imaris software. The Pearson correlation coefficient (PCC) revealed no correlation/signal overlap between both particles for all co-exposure conditions.

As we expected a synergistic effect of LPS-pretreated macrophages for the uptake of 920-nm SiO_2_-Cy5 and 59-nm SiO_2_-BDP FL particles (sequential and simultaneous exposures), we examined the particle colocalization (i.e., functional relationship) using Imaris software (version 9.5). The data is presented as Pearson correlation coefficient (PCC) values (r) analyzed for each co-exposure condition. The PCC values range from −1 to +1, where +1 indicates a total positive linear correlation, 0 no linear correlation, and −1 a total negative linear correlation. We hypothesized that 920-nm SiO_2_-Cy5 and 59-nm SiO_2_-BDP FL particles may colocalize within macrophages and that there could be differences in colocalizations between different exposure conditions. However, the PCC showed no colocalization of the two particles in LPS prestimulated and unstimulated exposures, indicating differential intracellular localization and no synergistic effect (Figure 6).

To estimate the intracellular colocalization of 59-nm SiO_2_-BDL FL and 920-nm SiO_2_-Cy5 with lysosomes, additional experiments with LysoTracker^TM^ were performed. Confocal laser scanning micrographs were used for a PCC analysis in Imaris software and are represented in Figure S4.

In unstimulated cells, 59-nm SiO_2_-BDP NPs showed a higher colocalization with lysosomes (PCC: 0.35–0.38) compared to 920-nm SiO_2_-Cy5 particles (PCC: 0.02–0.04). Similar observations were confirmed for LPS prestimulated cells: a profound colocalization between 59-nm SiO_2_-BDP NP and lysosomes was found (PCC: 0.24–0.35), whereas only a few colocalization events between 920-nm SiO_2_-Cy5 particles and lysosomes (PCC: −0.03–0.06) were observed. The PCC values were comparable between different co-exposure conditions for both unstimulated and LPS prestimulated cells.

### 3.7. Increased Uptake of Both SiO_2_ Particles after LPS Prestimulation in J774A.1 Macrophages

**Figure 7:**
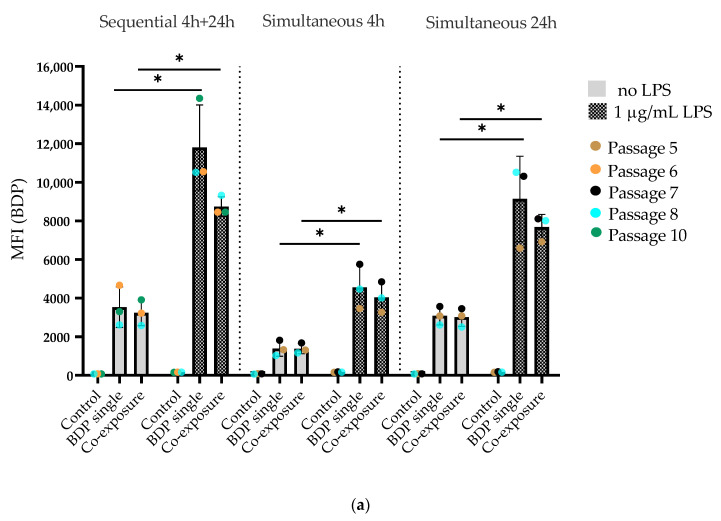

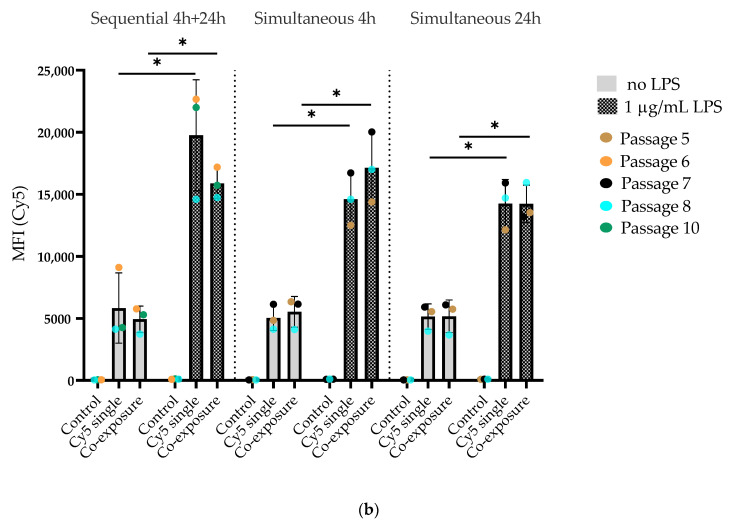
Flow cytometry data showing the uptake of SiO_2_ particles in J774A.1 macrophages. Cells were prestimulated with 1-µg/mL LPS for 24 h and then exposed to a single particle type or a combination of 59-nm SiO_2_-BDP FL and 920-nm SiO_2_-Cy5 particles. For comparison, we used unstimulated cells exposed only to SiO_2_ particles in the same manner. The particles were administered sequentially or simultaneously for different time points (4 h and 24 h). (**a**) Median fluorescence intensity (MFI) of the BDP FL signal. (**b**) Median fluorescence intensity (MFI) of the Cy5 signal. Data show a significant increase (* *p* < 0.05) in the MFI of the BDP FL and Cy5 signals after LPS prestimulation. No significant difference in the MFI was observed between the single- and co-exposure conditions. Control cells were stained with DAPI and were not exposed to SiO_2_ particles.

The uptake of SiO_2_ particles by J774A.1 macrophages was quantified by flow cytometry (Figure 7). BDP FL and Cy5 fluorescent signals coming from 59-nm SiO_2_-BDP FL NPs and 920-nm SiO_2_-Cy5 particles are represented separately as Figure 7a,b, respectively. We found that unstimulated macrophages internalized 59-nm SiO_2_-BDP FL NPs to a similar extent under single- and co-exposure conditions, as shown by comparable values for the median fluorescence intensity (MFI) of the BDP FL signal: 3527 vs. 3240 for sequential, 1389 vs. 1383 for 4-h simultaneous, and 3088 vs. 3013 for 24 simultaneous co-exposures (Figure 7a). Similarly, the MFI of the Cy5 signal were comparable between single- and co-exposures: 5829 vs. 4933 for sequential, 5036 vs. 5531 for 4 h simultaneous, and 5145 vs. 5168 (Figure 7b). The gating strategy is explained in Figure S5.

When the macrophages were prestimulated with 1-µg/mL LPS for 24 h and post-exposed to SiO_2_ particles (sequential and simultaneous), the MFI values increased approximately three times for both the BDP FL and Cy5 signals compared to unstimulated cells. This indicates elevated uptakes of both SiO_2_ particles into macrophages after LPS prestimulation. Similar to the observations in the unstimulated group, we did not observe significant differences in the MFI between the single- and co-exposure conditions in the LPS prestimulated group. In addition to MFI data, information on the percentages of live cells taken into a MFI analysis are shown in Figure S6.

## 4. Discussion

Understanding the behavior of different co-exposed particles in the human body is important for biomedical applications of these particles, as well as for their possible exposure to humans via the use of NP-containing products. We investigated the uptake of different-sized SiO_2_ particles, i.e., micron-sized and nanosized, in J774A.1 macrophages prestimulated with LPS for 24 h to mimic an inflamed environment.

First, we evaluated the cytotoxic effects of the SiO_2_ particles under single- vs. co-exposure conditions in unstimulated and LPS prestimulated cells. For most conditions, no cytotoxic effects were observed. A moderate but significant increase in LDH release was only observed for LPS prestimulated cells exposed simultaneously to both particles for 24 h. This can be explained by a cumulative cytotoxic effect of both the tested SiO_2_ particles and LPS added to the macrophages. Similar observations were published by Chun-Feng et al. [17], where the apoptosis of A549 cells was significantly increased after a combined exposure to lead (Pb) and nano-SiO_2_ for 24 h compared to either Pb or nano-SiO_2_ alone. The absence of a higher LDH release was observed at 4 h in comparison to the other exposure scenarios. As shown by other studies, the leakage of LDH increases continuously up to 24 h [36]. In addition, we did not observe a significant increase in LDH release when particles were added to the cells sequentially. This indicates that a simultaneous stimulation with two particles for 24 h causes a higher cell burden compared to the subsequent exposure to the same two particles. We assume that the different exposure conditions contribute to the cytotoxic effects.

After LPS prestimulation, we showed that a large proportion of cells change their morphology. As observed by Kim et al. [37], these changes could be related to rearrangements of actin filaments as a response to inflammatory stimuli (e.g., the formation of lamellipodia and filopodia). Such a mechanism is required for cell adhesion and migration to the sites of inflammation. We assumed that these changes could also be associated with cells phagocytic/endocytic activity and upregulated expressions of phagocytosis or endocytosis-related receptors on the cell surface. To test this hypothesis, we carried out flow cytometry studies to investigate if LPS prestimulated cells take up more SiO_2_ particles compared to the unstimulated group. The uptake of both particle types was enhanced in LPS prestimulated cells compared to the unstimulated control, as confirmed by three-times higher MFI values. These results are in agreement with previously published data [38,39], which reported an increased uptake of silica particles in M1 cells (IFN-γ/LPS-induced) when compared to M2 cells (IL-4-induced) using the murine RAW264.7 cell line. The increased uptake of silica by M1 macrophages was expected, as they are involved in the phagocytosis of foreign materials and pathogens. After polarization with LPS, M1 macrophages start expressing specific proteins essential for foreign particle recognition and phagocytosis [40]. However, other studies [30,31] showed higher uptakes of different-sized silica NPs (26 nm and 41 nm) in M2 macrophages while stimulating them with LPS/IFN-γ to generate the M1 phenotype and IL-10 to generate the M2 phenotype. These data suggest that different particle properties (e.g., size), as well as the cell type and interaction with the cellular environment, influence their uptake and the cellular behavior. We observed no significant differences between the single- and co-exposure conditions, i.e., sequential and simultaneous, on the particle uptake. Therefore, we assume that one SiO_2_ particle type does not facilitate the uptake of the other by inducing a crosstalk of endocytosis pathways and, thus, synergistic effects. These results are interesting, since it has been shown by other studies that the co-exposure of cells to different particles might act as an uptake accelerant [18,21]. Again, we assume that the discrepancies arise due to variations in the particle size, materials, and co-exposure conditions, and we have used very different sizes, i.e., 59-nm SiO_2_ NPs and 920-nm SiO_2_ particles.

To evaluate the effect of LPS on J774A.1 polarization, we performed an ELISA assay to determine the TNF-α (a specific marker of M1 macrophages) production. Twenty-four hours after LPS exposure, we observed a significant increase in TNF-α in the cell supernatant. This is in agreement with other publications [30,31,39,41] investigating the endocytosis of different-sized SiO_2_ NPs by macrophages while stimulating them with different cytokines. They demonstrated that, indeed a prestimulation with LPS and/or IFN-γ generated the M1 phenotype. When the stimulus was removed and the cells were exposed to SiO_2_ particles sequentially and simultaneously, we still could detect TNF-α in the cell supernatant but to a significantly lower extent, indicating that the inflammatory response is reduced. We decided not to keep LPS in the cell culture medium upon exposure to the particles, as this might interact with the particle surface, as shown elsewhere [42]. We did not observe any significant differences in TNF-α secretion between the single- and co-exposure conditions, indicating that the co-exposure to different-sized particles does not enhance the inflammatory response compared to individual particle exposures.

The uptake and intracellular distribution of both types of SiO_2_ particles were confirmed using confocal laser scanning microscopy. The corresponding z-stack images confirmed that the particles localized inside the cells and were not merely attached to their surfaces. To assess if the different-sized SiO_2_ particles were located in the same vesicles and if their distributions changed after LPS prestimulation, we analyzed the PCC, which assumes that two imaging targets are functionally related and might exist in the same region [43]. We could show that the different-sized SiO_2_ particles were not colocalized either in LPS prestimulated or in unstimulated macrophages. We assume that SiO_2_ particles might be located in different intracellular compartments in J774A.1 macrophages. In order to estimate the particle distribution within cellular compartments (i.e., lysosomes), additional experiments with LysoTracker^TM^ were performed. In both unstimulated and LPS prestimulated cells, we observed that 59-nm SiO_2_-BDP NP show an increased colocalization with lysosomes, as also observed by Schütz et al. [44]. On the other hand, 920-nm SiO_2_-Cy5 particles co-localized much less with lysosomes. A possible explanation is that LysoTracker^TM^ probes are weakly basic amines that selectively accumulate in cellular compartments, such as lysosomes with low internal pH (~4.5–5.0). The mechanisms of LysoTracker^TM^ accumulations in acidic vesicles involve molecule protonation in the interior of an acidic vesicle [45]. It has been shown that phagosomes formed specifically by M1 macrophages remained in the neutral range, with an average pH of 7.55 [46]. The reason for the higher colocalization between 59-nm SiO_2_-BDP and lysosomes compared to the moderate colocalization between 920-nm SiO_2_-Cy5 particles and lysosomes can be attributed to the different intracellular localizations of the SiO_2_ particles. SiO_2_-Cy5 particles (920 nm) might be localized in the phagosomes, whereas 59-nm SiO_2_-BDP NPs reached the lysosomes.

In summary, we could show that the uptake of silica NPs is enhanced in inflamed macrophages but not when co-exposed with micron-sized silica particles. Our data suggest that the interaction of NPs with inflamed macrophages should be taken into consideration when investigating the safety and efficacy of NPs for biomedical applications.

## Figures and Tables

**Table 1:** Representation of the main physicochemical characteristics of silica (SiO_2_) particles.

Particles	TEM ^6^	DLS ^7^				c ^5^ (mg/mL)
		Milli-Q			cRPMI	
	d_c_^1^ (nm)	d_h_^2^ (nm)	PDI ^3^	Ζ ^4^ (mV)	d_h_^2^ (nm)	
SiO_2_-Cy5	920 ± 88	908 ± 13	0.03	−44 ± 1	931 ± 19	143
SiO_2_-BDP FL	59 ± 6	76 ± 1	0.16	−52 ± 2	87 ± 6	9

^1^ core diameter, ^2^ hydrodynamic diameter measured after particle incubation at 37 °C for 24 h, ^3^ polydispersity index, ^4^ zeta potential, ^5^ concentration, ^6^ transmission electron microscopy, ^7^ dynamic light scattering, SiO_2_-Cy5: silica Cyanine 5, SiO_2_-BDP FL: silica BODIPY^®^-fluorescein, and cRPMI: Roswell Park Memorial Institute complete cell culture medium.

## Supplementary Materials

The following are available online at https://www.mdpi.com/2073-4409/9/9/2099/s1: Figure S1: Characterization of the SiO_2_ particles. Figure S2: Representative light microscopy images of J774A.1 macrophages in different cell passages. Figure S3: The intracellular localization of the silica particles after 4-h simultaneous co-exposures. Figure S4a: Intracellular localization of the SiO_2_ particles within lysosomes in unstimulated J774A.1 macrophages. Figure S4b: Intracellular localization of the SiO_2_ particles within lysosomes in LPS prestimulated J774A.1 macrophages. Figure S5: Gating strategy for the flow cytometry analysis. Figure S6: Analysis of the flow cytometry data by the percentage of live cells.
