# Supplementary material for: Increased Uptake of Silica Nanoparticles in Inflamed Macrophages but Not upon Co-Exposure to Micron-Sized Particles
Source: Cells. 2020 Sep 15;9(9):2099. doi: 10.3390/cells9092099 (PMC7564500; doi:10.3390/cells9092099)
Supplement: Supplementary file 1 [file cells-09-02099-s001.zip › Supplementary materials_accepted/Supplementary Materials_accepted.pdf]

cells

MDPI

Supplementary Materials

Increased uptake of silica nanoparticles in inflamed macrophages but not upon co-exposure to micron-sized particles

Eva Susnik[1], Patricia Taladriz-Blanco[1], Barbara Drasler[1], Sandor Balog[1], Alke Petri-Fink[1,2] and Barbara Rothen-Rutishauser[1]*

[1] Adolphe Merkle Institute, University of Fribourg, Chemin des Verdiers 4, 1700 Fribourg, Switzerland; eva.susnik@unifr.ch (E.S.); barbara.drasler@unifr.ch (B.D.); sandor.balog@unifr.ch (S.B.); patricia.taladrizblanco@unifr.ch (P.T.-B.)
[2] Department of Chemistry, University of Fribourg, Chemin du Musée 9, 1700 Fribourg, Switzerland; alke.fink@unifr.ch (A.P.-F.)
* Correspondence: barbara.rothen@unifr.ch (B.R.-R.); Tel.: +41 26 300 95 02

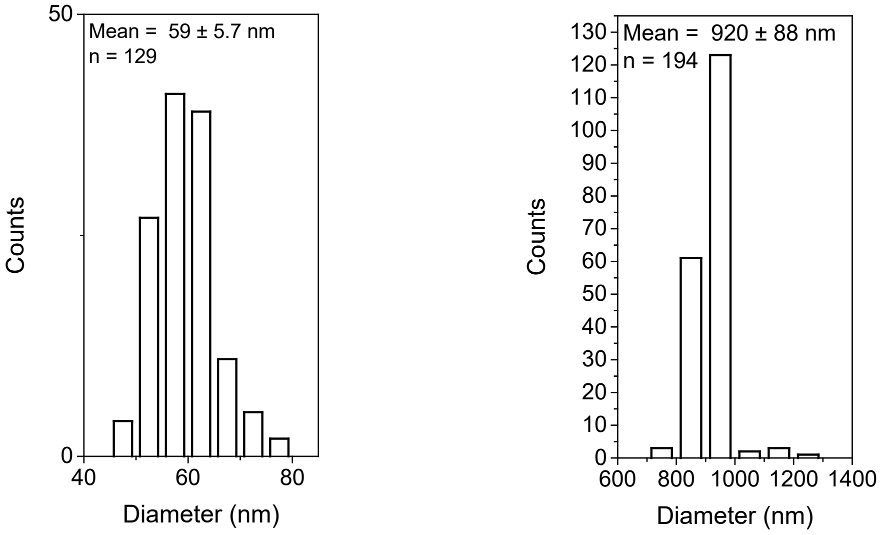

(**a**) (**b**)

Figure S1. Characterization of the SiO_2 particles**.** Size distribution, determined by transmission electron microscopy (TEM) and presented as histograms. (**a**) Size distribution of 59 nm SiO_2-BDP FL nanoparticles. (**b**) Size distribution of 920 nm SiO_2-Cy5 microparticles. Statistical analysis and graphical presentations were performed using the software Origin 2016 (OriginLab Corporation, Northampton, MA, USA).

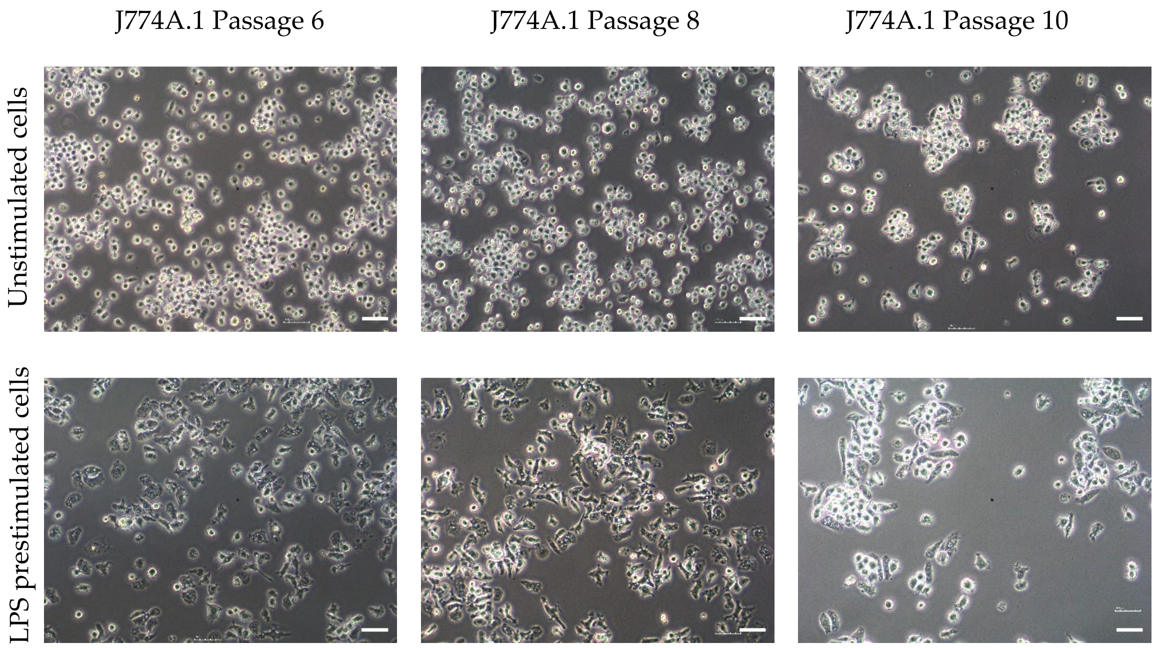

Figure S2. Representative light microscopy images of J774A.1 macrophages in different cell passages. **Upper pannels:** Unstimulated macrophages. **Lower pannels:** Macrophages 24 h after stimulation with 1 μg/mL LPS. After stimulation, the change in cell morphology (larger cells with more protrusions) is visible in all cell passages in comparison to more round unstimulated cells. Scale bar: 100 μm.

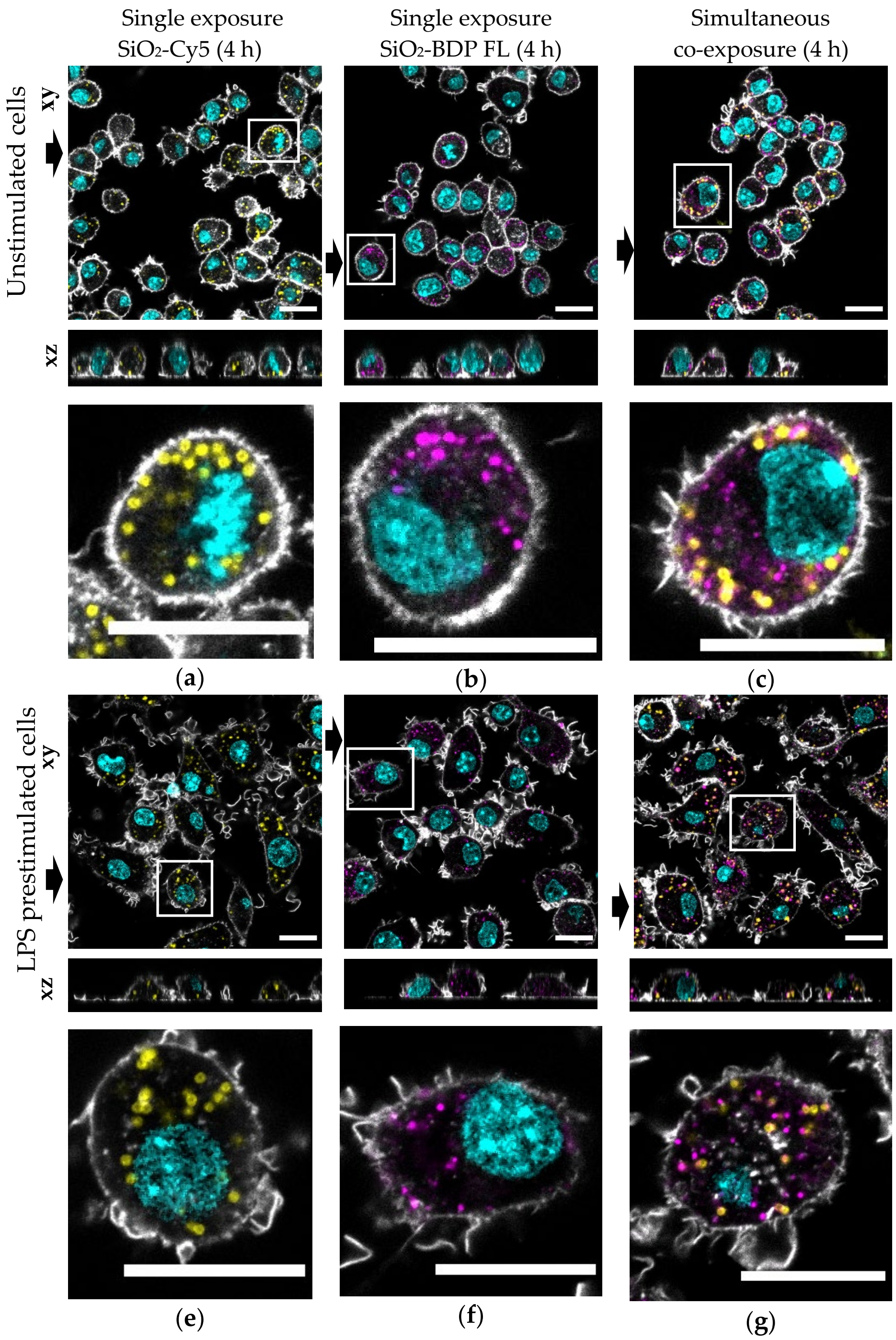

Figure S3. The intracellular localization of the silica particles after 4-h simultaneous co-exposures. Confocal laser scanning micrographs with the corresponding xz-projections demonstrate the uptake of 59 nm SiO_2-BDP FL NPs and 920 nm SiO_2-Cy5 particles in J774A.1 macrophages. **Upper panels (**a-c**):** Cells exposed to single SiO_2-Cy5 particles (yellow), single SiO_2-BDP FL NPs (magenta) or combination of SiO_2 particles simultaneously for 4 h. **Bottom panels** (e-g**):** Cells were prestimulated with 1 µg/mL LPS for 24 h and then post-exposed to single SiO_2-Cy5 particles, single SiO_2-BDP FL NPs or combination of both particles simultaneously for 4 h. F-actin was stained as cytoskeleton marker using a rhodamine-phalloidin conjugate (grey), and the nucleus was stained using 4′,6-diamidino-2-phenylindole (DAPI) (cyan). The black arrows represent the position of the xz-projection (at the bottom), showing the intracellular localization of particles. Zoom-in images (shown under each representative image) clearly demonstrate the cellular distribution of two types of SiO_2 particles. Scale bar: 20 µm.

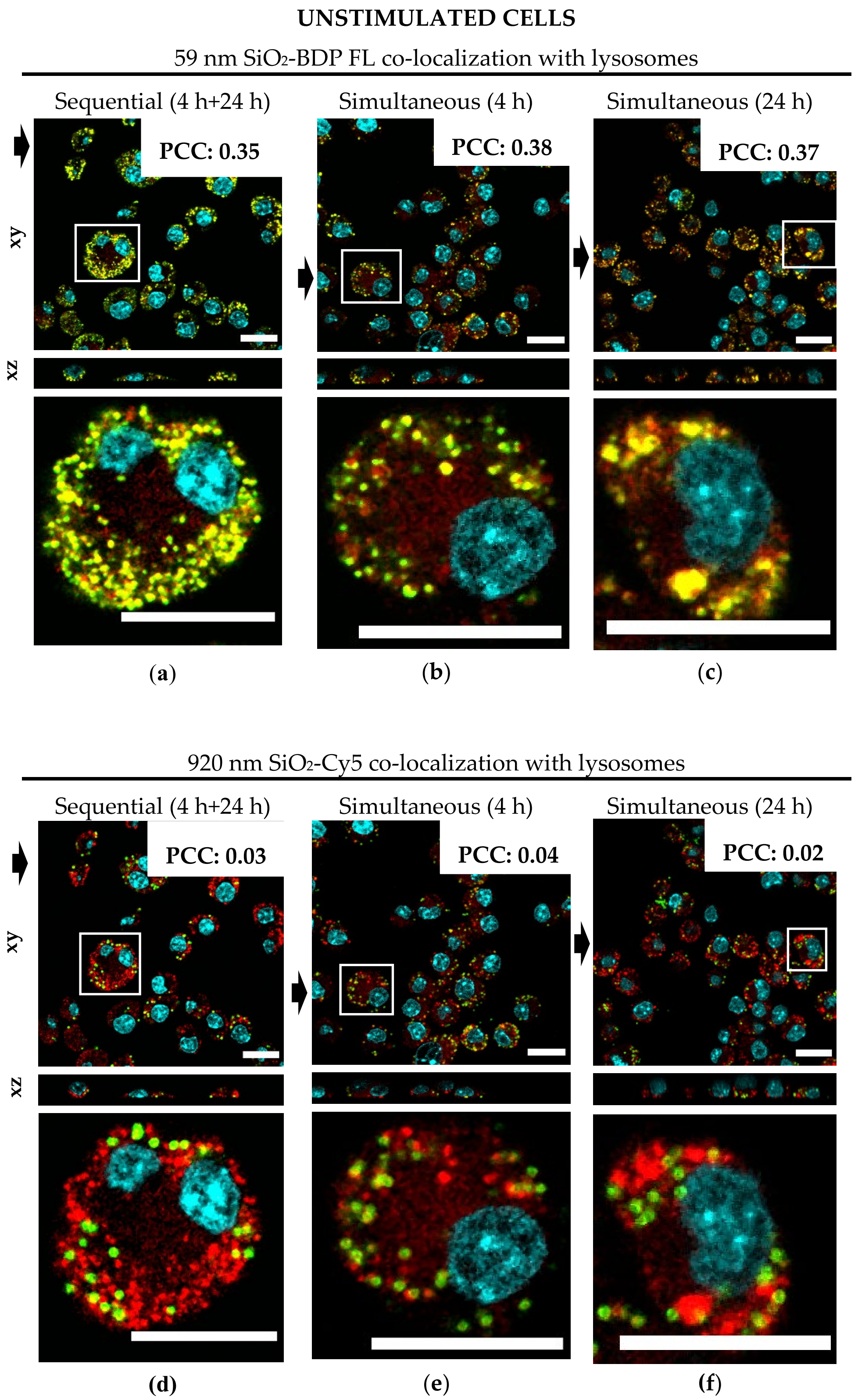

Figure S4a. Intracellular localization of the SiO_2 particles within lysosomes (LysoTracker™ Red) in unstimulated J774A.1 macrophages. Cells co-exposed to SiO_2 particles sequentially and simultaneously for 4 h and 24 h. (**a-c**) Co-localization between 59 nm SiO_2-BDP FL (green) and lysosomes (red). (**d-f**) Co-localization between 920 nm SiO_2-Cy5 (green) and lysosomes (red). Co-localization appears in yellow. Pearson correlation coefficient (PCC) is annotated in the top right corner. The black arrows represent the position of the xz-projection. Scale bar: 20 μm.

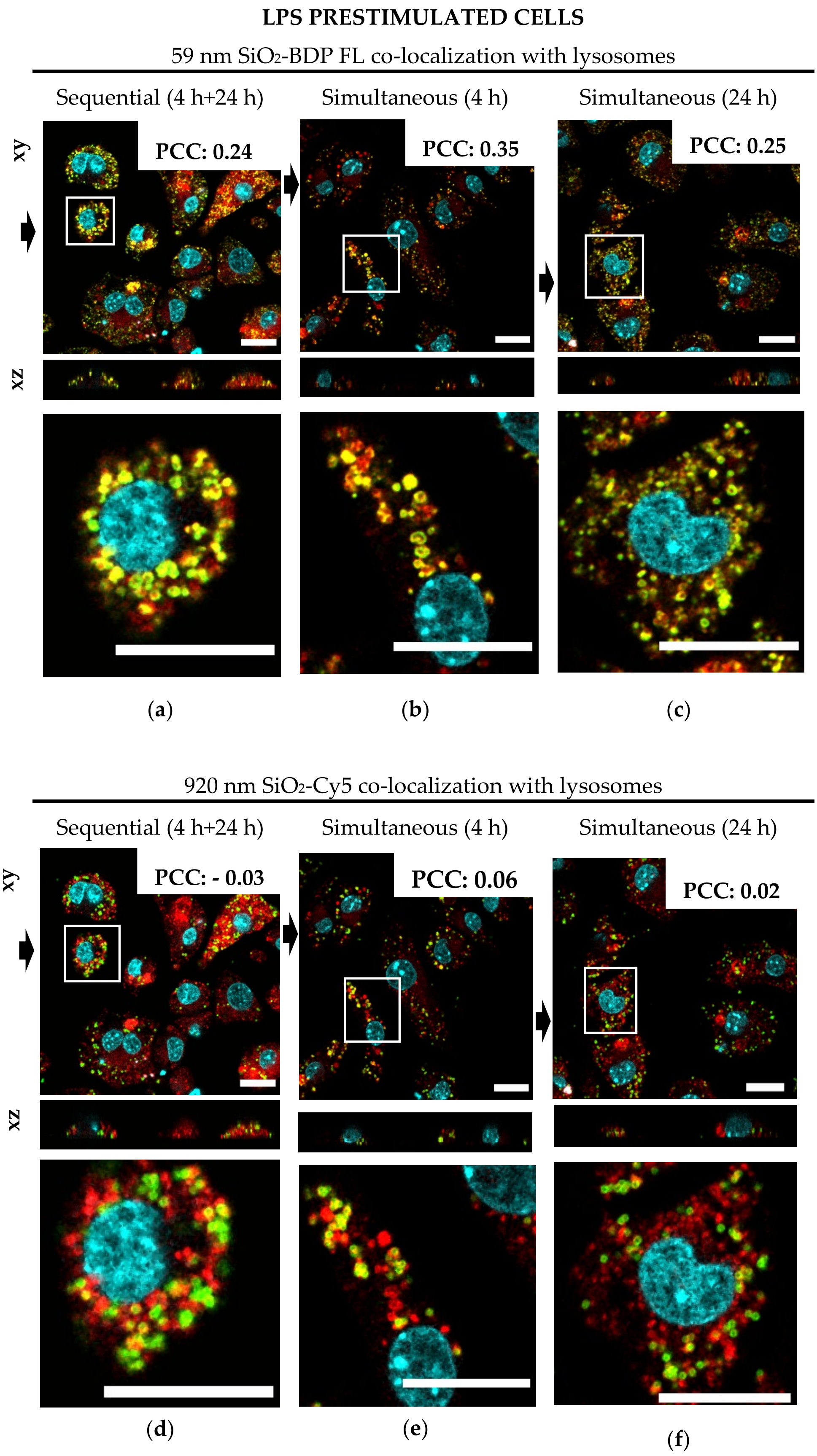

Figure S4b. Intracellular localization of the SiO_2 particles within lysosomes (LysoTracker™ Red) in LPS prestimulated J774A.1 macrophages. Cells co-exposed to SiO_2 particles sequentially and simultaneously for 4 h and 24 h. (**a-c**) Co-localization between 59 nm SiO_2-BDP FL (green) and lysosomes (red). (**d-f**) Co-localization between 920 nm SiO_2-Cy5 (green) and lysosomes (red). Co-

localization appears in yellow. Pearson correlation coefficient (PCC) is annotated in the top right corner. Scale bar: 20 μm.

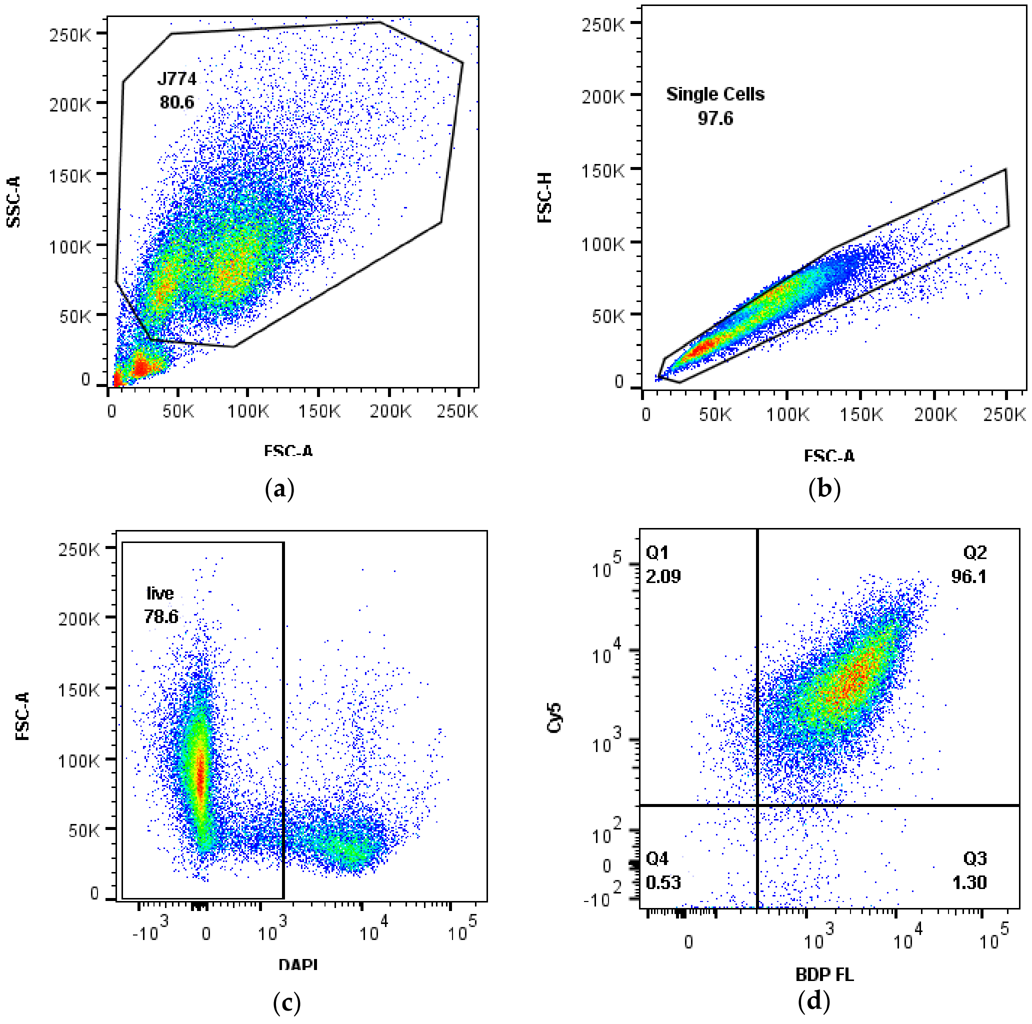

Figure S5. Gating strategy for flow cytometry analysis. Data analysis was performed in FlowJo software (Version 10.6.1, Tree Star, USA). (**a**) Forward and side scatter gates were set to exclude cell debris, possibly remained in the suspension. (**b**) Events were gated for doublets exclusion (FSC-A x FSC-H). (**c**) According to DAPI signal, cells were discriminated to live and dead, where DAPI negative events were considered alive. (**d**) To distinguish between Cy5 and BDP FL positive fluorescent signals, the quadrant gating has been set accordingly.

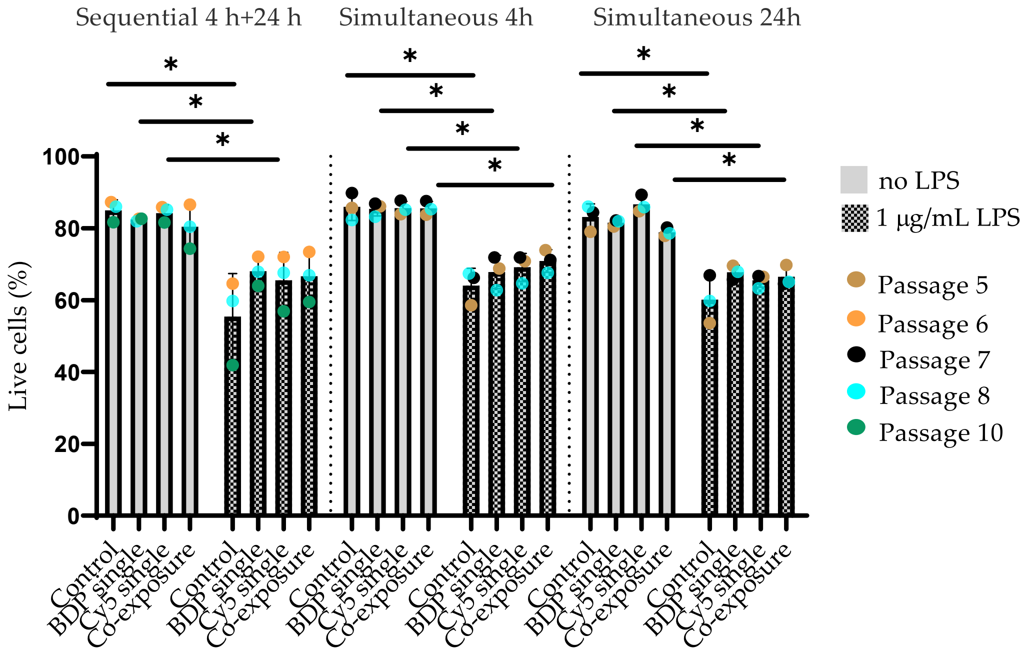

58 **Figure S6.** Analysis of flow cytometry data by percentage of live cells. Data shows the percentage of
59 living J774A.1 macrophages (events), where DAPI negative (live) cells were selected. This cell
60 population was then used for further analysis with specific fluorophores. Data shows comparison
61 between live cells of untreated control, cells exposed only to SiO_2-BDP (BDP single) or SiO_2-Cy5 (Cy5
62 single) and cells exposed to both particles. Data is compared among different experimental conditions
63 (sequential, simultaneous 4 h and simultaneous 24 h) and among LPS-stimulated and unstimulated
64 cells. Percentage of live cells was significantly lower in LPS-stimulated cells, compared to
65 unstimulated as indicated by *$p<0.05$ (GraphPad Prism; unpaired t-test).
